# Supplementary material for: Complexome profiling of the Chlamydomonas psb28 mutant reveals TEF5 as an early PSII assembly factor
Source: Plant Cell. 2025 Mar 17;37(6):koaf055. doi: 10.1093/plcell/koaf055 (PMC12164590; doi:10.1093/plcell/koaf055)
Supplement: koaf055_Supplementary_Data [file koaf055_supplementary_data.zip › psb28_interactive.zip!/plots/intensity/qualityPlots/peptides/petB_Cre-1.g2717017.t1.p1.html]

### Cre-1.g2717017.t1.p1

Trivial name: petB  
SeqCoverage [median]: 38.60  
Mass: 24.16 kDa  
  
 

| Sequence | MissedCleavage | Mass [Da] | wtIntensity | muIntensity | Score |
| --- | --- | --- | --- | --- | --- |
| GGVGVGQATLTR | 0 | 1114.61 | 281758.11 | 444886.85 | 192.12 |
| IVTGVPDAIPGVGGFIVELLR | 0 | 2121.22 | 7506.61 | 0.00 | 99.41 |
| LEIQAIADDITSK | 0 | 1415.75 | 256508.38 | 557580.65 | 268.98 |
| SKVYDWFEER | 1 | 1357.63 | 1640.60 | 2459.07 | 47.56 |
| VYDWFEER | 0 | 1142.50 | 93399.36 | 109311.05 | 174.27 |
| VYLTGGFK | 0 | 883.48 | 114422.78 | 287356.78 | 158.12 |
| VYLTGGFKRPR | 2 | 1292.74 | 0.00 | 2587.12 | 84.17 |
|  |  |  | 755235.8 | 1404181.5 |  |
